# Identification of PD-1–PD-L1 blockade epitopes in vitro utilizing porcine immunoglobulin and heterologous Fc-fused protein

**DOI:** 10.1186/s13567-025-01674-x

**Published:** 2025-11-27

**Authors:** Yuwen Dong, Xin Li, Zuxin Gong, Chenchen Liu, Jiaqi Dai, Shuhuai Shen, Zhen Yang, Gongguan Liu

**Affiliations:** Key Laboratory of Animal Diseases Diagnostic and Immunology, Ministry of Agriculture, MOE International Joint Collaborative Research Laboratory for Animal Health & Food Safety, College of Veterinary Medicine, Nanjing Agricultural University, Nanjing, 210095 China

**Keywords:** Programmed death 1, programmed cell death ligand 1, monoclonal antibody, epitopes, blockade

## Abstract

Programmed death 1 (PD-1) and its ligand, programmed death ligand 1 (PD-L1), function as pivotal immune checkpoints. Numerous studies have demonstrated the association between the malignant progression of various swine diseases and aberrant expression of PD-1 and PD-L1, hence the development and screening of high-affinity porcine PD-1 and PD-L1 monoclonal antibodies (mAbs) holds substantial significance for advancing research and therapeutic interventions. In this study, we produced porcine PD-1 and PD-L1 mAbs which exhibited robust reactivity in western blot (WB), indirect immunofluorescence assay (IFA), and flow cytometry (FCM). The newly identified B-cell epitope ^90^GRDPRFHVTPL^100^ and ^185^REEKLFNVTST^195^ of PD-1 and PD-L1 mAbs were linear and surface-exposed as illustrated by WB and structure analysis. Comparative sequence analysis demonstrated that the PD-L1 epitope is highly conserved across species, whereas the PD-1 epitope exhibits lower interspecies conservation. In addition, the blocking efficacy of the two PD-1 mAbs and six PD-L1 mAbs was predicted low via molecular docking. To further evaluate the blocking efficacy, we generated a flow cytometry-based assay by using a porcine PD-L1-rabbit Fc fusion protein, expressed via a eukaryotic system. In agreement with the prediction, our in vitro data demonstrated a blocking rate below 4% compared with the IgG group for PD-1 mAbs and PD-L1 mAbs. In summary, we herein generated porcine PD-1 and PD-L1 mAbs recognizing unreported B-cell epitopes, and established a reliable method for identifying the nonblocking mAb and epitopes, which may facilitate the development of novel diagnostic approaches and therapeutic agents.

**Supplementary Information:**

The online version contains supplementary material available at 10.1186/s13567-025-01674-x.

## Introduction

Programmed death 1 (PD-1) is a critical immunoregulatory molecule involved in immune tolerance and T cell exhaustion, with predominant expression observed on activated T cells, B cells, dendritic cells (DCs), and macrophages. Its primary ligand, programmed death ligand 1 (PD-L1), is extensively expressed on tumor cells, immune cells, epithelial cells, and endothelial cells. Under physiological conditions, the PD-1–PD-L1 axis serves as a negative regulator of T cell activation. The binding of PD-1 to PD-L1 potentiates immunosuppressive signaling and promotes antigen-specific immune tolerance, thereby mitigating excessive tissue damage and maintaining systemic self-tolerance. However, neoplastic cells and certain pathogens exploit this coinhibitory pathway by upregulating PD-L1 expression, which subsequently engages PD-1^+^ T cells, resulting in T cell apoptosis, functional exhaustion, and immune microenvironment remodeling. Accumulating evidence indicates that the PD-1–PD-L1 signaling pathway is significantly implicated in infectious diseases caused by the most prevalent pathogens affecting the pig industry, namely porcine reproductive and respiratory syndrome virus (PRRSV), classical swine fever virus (CSFV), and porcine circovirus 2 (PCV2).

Consistent upregulation of PD‑1–PD‑L1 expression has been observed in porcine tissues or cells infected with CSFV or PCV2 [1–4]. In addition, activation of other immune checkpoint molecules such as CD276 and LAG3 were also detected. In both in vivo and in vitro models of PRRSV infection, the PD‑1–PD‑L1 molecules were significantly upregulated, accompanied by marked T‑cell exhaustion. Increasing findings support that the upregulation of negative checkpoint molecules is closely related to disease progression during PRRSV infection [5, 6]. These findings support that PD‑1–PD‑L1 play a pivotal role in the swine infectious diseases and represent potential targets for regulating disease progression.

Owing to its immunosuppressive role, aberrant PD-1–PD-L1 activation serves as an early diagnostic biomarker for tumorigenesis and chronic viral infections. To date, PD-1–PD-L1-targeted therapies have been most extensively applied in the field of oncology. Ten *α*-PD-1 (camrelizumab, cemiplimab, dostarlimab, nivolumab, pembrolizumab, prolgolimab, sintilimab, toripalimab, tislelizumab, and zimberelimab) and three *α*-PD-L1 antibodies (atezolizumab, avelumab, and durvalumab) have been approved for clinical cancer therapy [7]. PD-1–PD-L1 axis has also demonstrated significant efficacy in controlling viral infections. Knockout of PD-1 or administration of PD-L1 antibodies effectively restores T-cell function and controls viral load in mice infected with lymphocytic choriomeningitis virus (LCMV) [8, 9]. Inhibition of PD-1 signaling has been shown to mitigate hepatitis B virus (HBV) infection by reversing T-cell exhaustion, augmenting interferon-gamma (IFN-γ) production, and stimulating the proliferation of peripheral blood mononuclear cells (PBMCs) [10, 11]. In addition to their role as monotherapy, PD-1–PD-L1 blocking antibodies can also enhance the immune efficacy of cancer vaccines and viral vaccines [12, 13]. A recent in vivo study demonstrated that upon treatment with a PD‑L1‑blocking antibody, IL‑2 production was markedly increased by PBMCs from pigs chronically infected with PRRSV, *Mycoplasma hyopneumoniae*, and PCV2, indicating the activation of the immune response [4]. These researches reinforce the therapeutic potential of PD-1–PD-L1 blockade strategies in viral infections. However, research and blocking antibody development targeting the PD-1–PD-L1 axis in veterinary medicine remain limited. Consequently, the development of monoclonal antibodies (mAbs) with PD-1–PD-L1 blocking activity represents a promising strategy for swine disease prevention and control.

In the present study, we generated two mAbs targeting PD-1 and six mAbs targeting PD-L1 from single B cells of vaccinated mice with recombinant PD-1 and PD-L1 protein. All mAbs possess excellent western blot (WB), immunofluorescence assay (IFA), and flow cytometry (FCM) reactivities. Moreover, two linear B-cell epitopes, ^90^GRDPRFHVTPL^100^ and ^185^REEKLFNVTST^195^, recognized by PD-1 mAbs and PD-L1 mAbs, respectively, were identified via WB. Based on these materials, blocking assay method for PD-1–PD-L1 antibodies has been developed by constructing fusion protein of PD-L1 and rabbit Fc, and the blocking effect of the mAbs was evaluated. The results showed that the PD-1 mAb 1G7D5 had 4% blockade ratios, and that of PD-L1 mAbs 1E3E3 and 1E3C3 were 2%, while there was no significant effect for other PD-1 and PD-L1 mAbs. In a word, these findings provided practical tools for the functional research of porcine PD-1 and PD-L1 proteins, establishing a blocking evaluation method for PD-1 and PD-L1 mAbs, which will contribute to the development of blocking antibodies against porcine PD-1 and PD-L1.

## Materials and methods

### Cells, plasmids, virus, and animals

Mouse myeloma cells (SP2/0), HEK-293 T, and HEK293F cells were kept in our laboratory. HEK-293 T cells were cultured in Dulbecco’s modified Eagle’s medium (DMEM) with 10% fetal bovine serum (FBS) in an incubator at 37 ℃ and 5% CO_2_. SP2/0 cells were cultured in RPMI-1640 (Gibco, NY, USA) with 15% FBS. *E. coli* strains DH5α and BL21 (DE3) were preserved in our laboratory. Vectors pcDNA3.1, pET-32a, pVAX1, and pGEX-4 T-1 were stored in our laboratory. ICR and BALB/c mice (6–8 weeks old, female) were procured from the Experimental Animal Center of Yangzhou University.

### Gene cloning, prokaryotic expression, and purification

Amplification extracellular region oligos of porcine PD-1 and PD-L1 genes were designed according to the PD-1 and PD-L1 genome sequence (GenBank accession number NM_001025221.1 and NM_001204379.1), and the *EcoR* I and *Xho* I digestion sites were added to the upstream and downstream of oligos, respectively. The primers were synthesized by Sangon Biotech Corporation (Shanghai, China), as shown in Table 1. The target products amplified using primers above, were then connected with pET-32a, and transformed into DH5α. Recombinant plasmids pET-32a-PD-1 or pET-32a-PD-L1 were identified by bacterial colony PCR. Similarly, PD-1 and PD-L1 fragment were inserted into the pcDNA3.1 vector using *EcoR* I and *Xho* I enzyme sites to conduct pcDNA3.1-PD-1 and pcDNA3.1-PD-L1 plasmids.

**Table 1:** **Primers for amplification and truncation of PD-1 and PD-L1**

Fragment	Amplification region		Name of primers	Sequences
PD-1	24–166 aa	sPD1-F		CCGCTCGAGTAAGTGGCCTTCGGGCCT
		sPD1-R		CGGAATTCCTCCTAGATGCCCCCAGCAGGC
PD-L1	19–237 aa	sPDL1-F		CCGCTCGAGCGTCTCCTCAAATTGTG
		sPDL1-R		CGGAATTCTTTACTATCACAGTTCCCA
PD-1	1–288 aa	PD1-F		CCAGTGTGGTGGAATTCATGGGGACCCCGC
		PD1-R		ACGTCGTATGGGTAGAGGGGCCAAGAGC
PD-L1	1–287 aa	PDL1-F		AACTTAAGCTTGGTACCATGAGGATATGTA
		PDL1-R		TGGTACGTCGTATGGGTACGTCTCCTCAAAT
PD-1-F1	24–100 aa	PD1-F1-F		GGATCCCCGGAATTCCTCCTAGATGCCCC
		PD1-F1-R		GTGATGGTGATGGTGCAGCGGCGTGACAT
PD-1-F2	90–166 aa	PD1-F2-F		GGATCCCCGGAATTCGGCCGGGACCCACG
		PD1-F2-R		GTGATGGTGATGGTGTAAGTGGCCTTCG
PD-L1-F1	19–133 aa	PDL1-F1-F		GGATCCCCGGAATTCTTTACTATCACAGTTC
		PDL1-F1-R		GTGATGGTGATGGTGTGGAGCATTGACTT
PD-L1-F2	123–237 aa	PDL1-F2-F		GGATCCCCGGAATTCTACAAGCGGATTAC
		PDL1-F2-R		GTGATGGTGATGGTGAGTCCTCTTTCTTG
PD-L1-F3	123–185 aa	PDL1-F3-F		GGATCCCCGGAATTCTACAAGCGGATTAC
		PDL1-F3-R		GTGATGGTGATGGTGTCTCTGGGAACTGG
PD-L1-F4	175–237 aa	PDL1-F4-F		GGATCCCCGGAATTCAGTGGCAAAACCA
		PDL1-F4-R		GTGATGGTGATGGTGAGTCCTCTTTCTTG
PD-L1-F5	123–195 aa	PDL1-F5-F		GGATCCCCGGAATTCTACAAGCGGATTAC
		PDL1-F5-R		GTGATGGTGATGGTGCGTGCTGGTCACATT
PD-L1-F6	123–205 aa	PDL1-F6-F		GGATCCCCGGAATTCTACAAGCGGATTAC
		PDL1-F6-R		GTGATGGTGATGGTGAATCTCATTAGTTGT
PD-L1-Fc	19–237 aa	PD-L1-Fc-F		CGCTGTGGCCGAGGCCTTTACTATCACAGTT
		PD-L1-Fc-R		GCCAGAACCGCCACCGCCAGTCCTCTTTCTT

After confirmed by bacterial colony PCR and sanger sequencing, the positive recombinant plasmids pET-32a-PD-1 and pET-32a-PD-L1 were transformed into *E. coli* BL21 (DE3) and cultured. When the OD_600_ of bacteria reached 0.6–0.8, 0.5 mM IPTG was added for induction for 6 h at 37 ℃. The supernatant and inclusion products of the induced bacteria were collected and identified by sodium dodecyl sulfate–polyacrylamide gel electrophoresis (SDS-PAGE) and western blot. Subsequently, the induced pET-32a-PD-1 and pET-32a-PD-L1 recombinant protein were dissolved by urea dialysis and then purified by high affinity Ni–NTA Resin (Genscript, Nanjing, China).

### Immunization of animals

The animal experiment protocol was approved by the Animal Care and Ethics Committee of Nanjing Agricultural University, Nanjing, China. The procedures were conducted according to the Guiding Principles for Biomedical Research Involving Animals.

Five female BALB/c mice (6–8 weeks old) were separately immunized with PD-1 and PD-L1 recombinant proteins. The immunization procedure was performed as follows. Recombinant proteins were mixed with Freund’s complete adjuvant in equal volumes and emulsified. The immunization was performed by subcutaneous injection of the dorsum at 100 µg per mouse. At 3 weeks after the first immunization, recombinant proteins were emulsified with Freund’s incomplete adjuvant in equal volume and injected at the same dose. The third immunization was repeated 3 weeks after the second immunization. Lastly, the shock immunization was performed 7 days later, and the antibody levels in the sera were measured by indirect enzyme-linked immunosorbent assay (ELISA) to select the mice with the highest antibody levels.

### Preparation of mAbs

After 3 days of shock immunization, the mice were euthanized to collect spleen cells. Then, counted SP2/0 cells and spleen cells were fused at a 7:1 ratio using PEG4000. The fused cells were seeded into 96-well plates containing RPMI-1640 medium supplemented with 15% FBS and 2% HAT (Solarbio, Beijing, China). After 10 days, the supernatant of 2% HAT was completely replaced with 2% HT (Solarbio). All culture supernatants of hybridoma cells were assessed by indirect ELISA after 48 h. Positive hybridoma cells were cloned at least three times using the limiting dilution approach. After at least 15 generations of expansion culture, the positively selected hybridoma cells that stably secrete antibodies were intraperitoneally injected into 8 week-old female BALB/c mice that had been presensitized with liquid paraffin, to harvest ascites. The titer of the ascites was identified via indirect ELISA and the specificity of mAbs was assessed by WB, IFA, and FCM. Finally, the heavy and light chain types of the mAbs were determined by a monoclonal antibody isotype identification kit (Proteintech, Wuhan, China).

### Indirect ELISA

The PD-1 or PD-L1 recombinant protein was coated in 96-well ELISA plates overnight at 4 ℃ at a density of 2 ng/mL (solution containing 0.1 M NaHCO_3_, 100 μL/well). Then, the plates were blocked with 100 μL 5% bovine serum albumin (BSA) per well at 37 ℃ for 1 h after the plates were washed three times with phosphate buffer containing 0.05% Tween-20 (PBST). And then, hybridoma cell culture supernatant, ascites, or serum were added into plates. After incubation at 37 ℃ for 1 h, the plates were washed three times with PBST and incubated with HRP-conjugated goat anti-mouse IgG (diluted at 1:3000 times) for 1 h at 37 ℃. After incubation, TMB was added for 20 min; the reaction was stopped with 2 M sulfuric acid, and the absorbance was measured at 450 nm to determine antibody binding.

### SDS-PAGE and western blot

The protein samples or cellular lysis were loaded to 12% SDS–polyacrylamide gels for electrophoresis. Gels were incubated with Coomassie brilliant blue to dye or transferred onto a nitrocellulose membrane (Cytiva, MA, USA). The membrane was blocked with 5% skimmed milk in TBST and then incubated with the appropriate primary antibodies at 4 °C overnight. After washing with TBST three times, the membrane was incubated with HRP-conjugated secondary antibodies at room temperature for 1 h and washed three times with TBST. All membranes were visualized with the ECL reaction buffer and imaged with a Tanon automatic chemiluminescence image analysis system according to the manufacturer protocols (Tanon, Shanghai, China).

### Indirect immunofluorescence assay and flow cytometry

The HEK293T cells transfected with pcDNA3.1-PD-1 or pcDNA3.1-PD-L1 were fixed with 4% paraformaldehyde and followed by permeabilization with 0.1% Triton X-100. Then, the nonspecific epitopes were blocked with 5% BSA diluted into PBS for 1 h and the primary antibodies (diluted 50 times) were incubated at 37 ℃ for 1 h. Finally, the fluorescence-conjugated secondary Alexa Fluor^™^ 488 or Alexa Fluor^™^ 594 antibody (Thermo Fisher, MA, USA) (for 1:2000 dilution) was incubated for 1 h in darkness. Following three washes, the signal was visualized with a confocal laser scanning microscope. Cells were analyzed using a CytoFLEX flow cytometer (Beckman Coulter, CA, USA).

### Transfection

HEK293T cells were maintained in DMEM supplemented with 10% FBS at 37 °C in 5% CO_2_. HEK293F cells were maintained in 293 Cell Culture Medium (Serum-free). Transfection was performed using polyethylenimine (PEI) for HEK293T and HEK293F cells according to the manufacture’s protocols. After 36 h post-transfection, the cells could be used for the following assays. A 3% SMS 293-SUPI was added into HEK293F cells after 12 h post-transfection, and the supernatant was collected for use.

### Antigenic epitope analysis

To truncate the PD-1 and PD-L1 proteins, we designed eight pairs of PCR oligos (Table. 1) and introduced an *EcoR* I digestion site at the 5′ end and an *Xho* I digestion site at the 3′ end. Two truncated overlapping PD-1 fragments and six truncated overlapping PD-L1 fragments were amplified by PCR and cloned into the pGEX-4T-1 vector, and recombinant truncated proteins were expressed in *E. coli* BL21 (DE3). These proteins were used for mAbs epitope mapping via WB.

### Biological information analysis

The amino acid sequences of PD-1 and PD-L1 from different species (*Sus_scrofa, Homo_sapiens, Chlorocebus_sabaeus, Bos_taurus, Mus_musculus, Capra_hircus, Canis_lupus_familiaris*) were assessed in NCBI [14]. Alignment analysis was performed using MEGA [15], and then, the conservation of PD-1 and PD-L1 epitopes among the different species was analyzed by Jalview software [16].

The protein structures of porcine PD-1 and PD-L1 were obtained from the UniProt, and uploaded to Alphafold3 [17] to get their spatial conformations and results of PD-1 and PD-L1 interaction. The spatial characteristics of the antigenic epitopes were analyzed by mapping the epitope positions to the predicted structural models via Pymol software. Meanwhile, the interaction sites of porcine of PD-1 and PD-L1 were visualized and the hydrophilic and hydrophobic properties of amino acids were analyzed by Pymol [16].

### PD-L1-Fc plasmid construction, eukaryotic expression, and purification

In order to obtain a protein that could specially bind to the PD-1 on the surface of cells for use in blocking assays, we conducted the PD-L1-Fc recombinant plasmid. The PD-L1-Fc plasmid consisted of PD-L1, rabbit IgG Fc fragment and a GGGS liner by using pVAX1 as vector. After bacterial colony PCR and Sanger sequencing, the PD-L1-Fc plasmid was transfected into 293F cells for eukaryotic expression. After 96 h post-transfection, the cell supernatant was collected by centrifugation for purification, which was similar to the method described above. The purified PD-L1-Fc protein was identified by sodium dodecyl sulfate–polyacrylamide gel electrophoresis (SDS-PAGE) and western blot.

### Blockade assay of mAbs

HEK293T cells were transfected with porcine pcDNA3.1-PD-1 plasmid firstly for 24 h. To determine the blocking effect of PD-1 mAbs, the cells were incubated with a control IgG or PD-1 mAbs for 1 h at 37 °C and then treated with PD-L1-Fc protein for another 1 h. After these, the cells were digested and collected for the FCM detection. To detect the blocking effect of PD-L1 mAbs, the cells were incubated with a control IgG or a precombined mixture of PD-L1 mAbs and PD-L1-Fc protein for 1 h. And then the cells were digested and collected for the FCM detection.

### Statistical analysis

Data were analyzed with GraphPad Prism 8 software. Data are expressed as the mean ± SD.

## Results

### Expression and purification of porcine PD-1 and PD-L1 proteins

**Figure 1:**
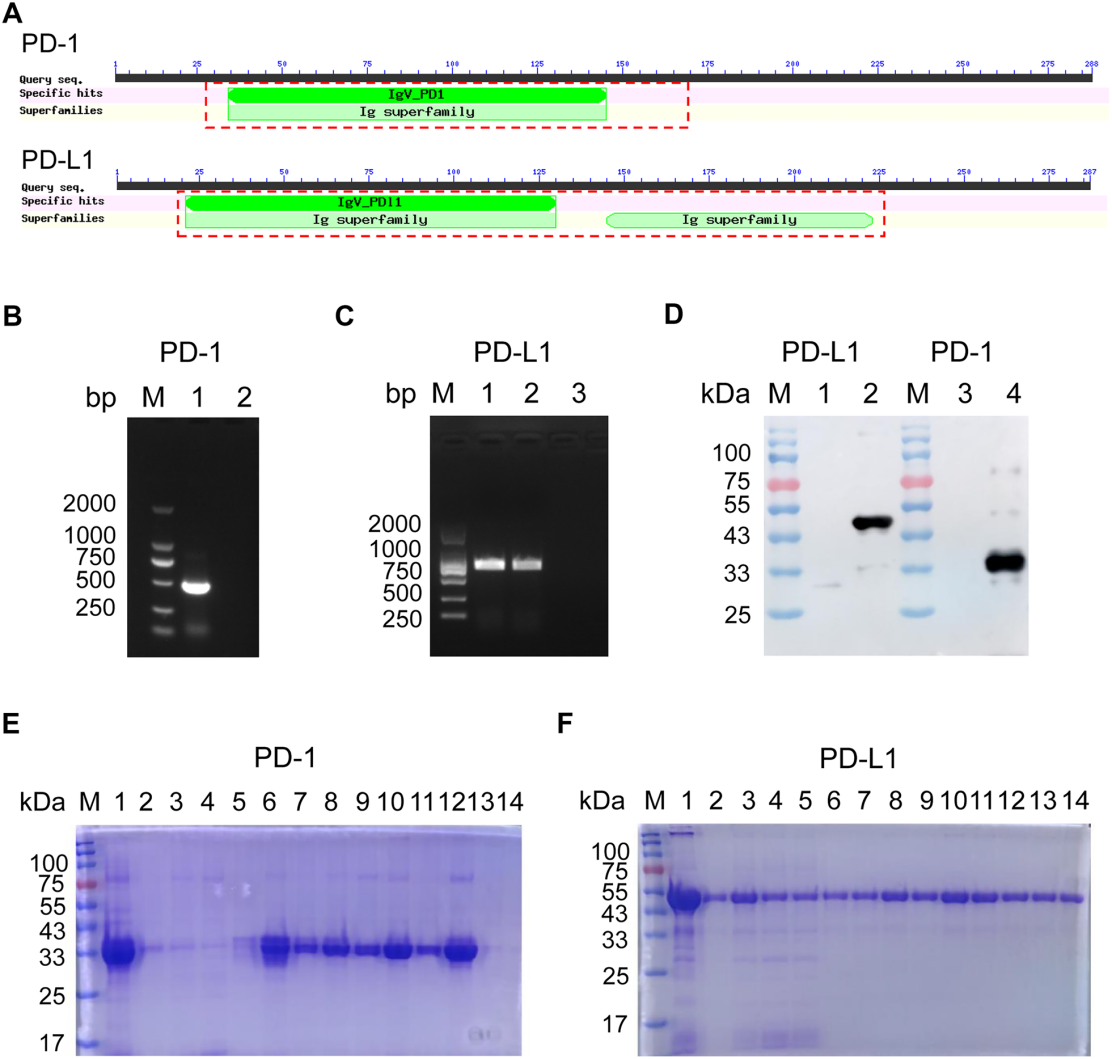
**Expression and purification of the His-tagged PD-1 and PD-L1 recombinant proteins**. **A** Domain structure of porcine PD-1 and PD-L1 proteins. The extracellular domain regions of porcine PD-1 and PD-L1 are highlighted with red boxes. **B** PCR amplification of porcine PD-1 extracellular region. M, DNA ladder; Line 1, PCR products of the extracellular zone porcine PD-1 gene; Line 2, Negative control. **C** PCR amplification of porcine PD-L1 extracellular region. M, DNA ladder; Line 1 and 2, PCR products of the extracellular zone of porcine PD-L1 gene; Line 3, Negative control. **D** Recombinant PD-1 and PD-L1 recombinant proteins were expressed using *E. coli* system under the treatment of 0.5 mM IPTG, and was analyzed by western blot using a mouse anti-His mAb. M, Protein Marker; Line 1 and 3, pET32a; Line 2, pET32a-PD-L1 recombinant protein; Line 4, pET32a-PD-1 recombinant protein. **E** and **F** Purified PD-1 and PD-L1 recombinant proteins were analyzed by SDS-PAGE. M, Protein Marker; Line 1, the supernatant after ultrasonication, urea dissolution, and centrifugation of the bacterial bodies; Line 2, the precipitate after ultrasonication, urea dissolution, and centrifugation of the bacterial bodies. Line 3–5, flowthrough fluid. Line 6–14, proteins by gradient elution.

According to the NCBI database, the porcine PD-1 extracellular domain ranges from amino acids 25 to 170, and the extracellular domain of porcine PD-L1 is situated from amino acids 19 to 237. The PD-1 extracellular domain comprises a single Ig-like V-type domain, whereas the PD-L1 extracellular domain contains both an Ig-like V-type and an Ig-like C2-type domain (Figure 1A). Recombinant PD-1 and PD-L1 proteins were expressed in vitro through PCR amplification, ligation, and subsequent expression in an *Escherichia coli* system (Figures 1B, C). As expected, the recombinant PD-1 protein exhibited a molecular weight of 33 kDa, and PD-L1 migrated at approximately 47 kDa (Figure 1D). The induced expression products were purified using a Ni-NTA agarose resin system, and Coomassie brilliant blue staining confirmed the isolation of highly pure recombinant proteins (Figures 1E, F).

### Determination of serum titer of immunized mice

**Figure 2:**
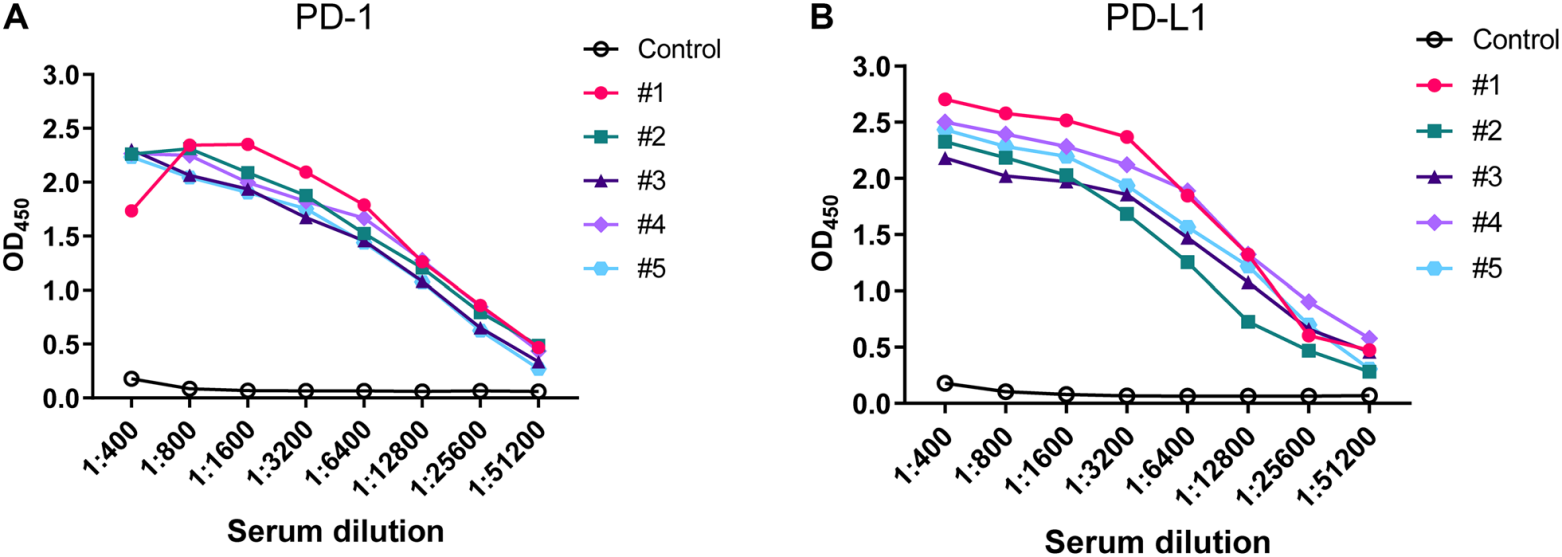
**Serum antibody titer of mice after triple immunized with PD-1 and PD-L1 recombinant proteins**. After the final booster immunization, serum was collected from the mice. Serial twofold diluted serum samples were tested by indirect ELISA with the purified recombinant PD-1 (**A**) or PD-L1 (**B**) protein for coating. The serum of unimmunized mice was tested in the same manner and served as the negative control. The antibody titers were represented as the absorbance value at OD_450_nm.

The serum antibody titer of mice immunized with PD-1 or PD-L1 recombinant proteins were quantified by indirect ELISA. Comparative analysis revealed significantly elevated antibody levels in immunized mice relative to nonimmunized controls (Figure 2). These data demonstrate that both recombinant proteins elicited robust humoral immune responses, confirming their immunogenicity in this model system.

### Preparation and identification of PD-1 and PD-L1 mAbs

Following mice immunization, cell fusion, and subcloning assay, two hybridoma cell clones (1G7D5 and 1C4G6) were identified by ELISA as producers of PD-1 antibodies. In parallel, six hybridoma cell clones (1E3E3, 3G6E6, 3D3F4, 1E3C3, 5D5E3, and 3B3E9) producing PD-L1 antibodies were also generated. The supernatant titers of the PD-1 hybridoma cell lines 1G7D5 and 1C4G6 remained stable at 1:25 600 and 1:102 400, respectively. The antibody titers secreted by PD-L1 hybridoma cell lines were consistently maintained between 1:25 600 to 1:102 400. To determine the antibody isotypes, an antibody-subtyping kit was employed. According to the data in Table 2, both PD-1 mAbs possess kappa light chains. The heavy chain of clone 1G7D5 belongs to the IgG class, whereas that of clone 1C4G6 is of the IgM class. All six PD-L1 mAbs are of the IgG isotype and likewise exhibit kappa light chains.

**Table 2:** **Titer and subtype of mAbs**

mAbs	Titers in cell culture supernatant				Titers in ascites	Heavy chain	Light chain
	P5	P10	P15	P20			
1C4G6	1:102 400	1:102 400	1:102 400	1:102 400	1:3 276 800	IgM	Kappa
1G7D5	1:25 600	1:25 600	1:25 600	1:25 600	1:6 553 600	IgG1	Kappa
1E3E3	1:102 400	1:102 400	1:102 400	1:102 400	1:6 553 600	IgG1	Kappa
3G6E6	1:102 400	1:102 400	1:102 400	1:102 400	1:3 276 800	IgG1	Kappa
3D3F4	1:102 400	1:102 400	1:102 400	1:102 400	1:6 553 600	IgG1	Kappa
1E3C3	1:51 200	1:51 200	1:51 200	1:51 200	1:6 553 600	IgG1	Kappa
5D5E3	1:25 600	1:25 600	1:25 600	1:25 600	1:819 200	IgG1	Kappa
3B3E9	1:25 600	1:25 600	1:25 600	1:25 600	1:1 638 400	IgG1	Kappa

**Figure 3:**
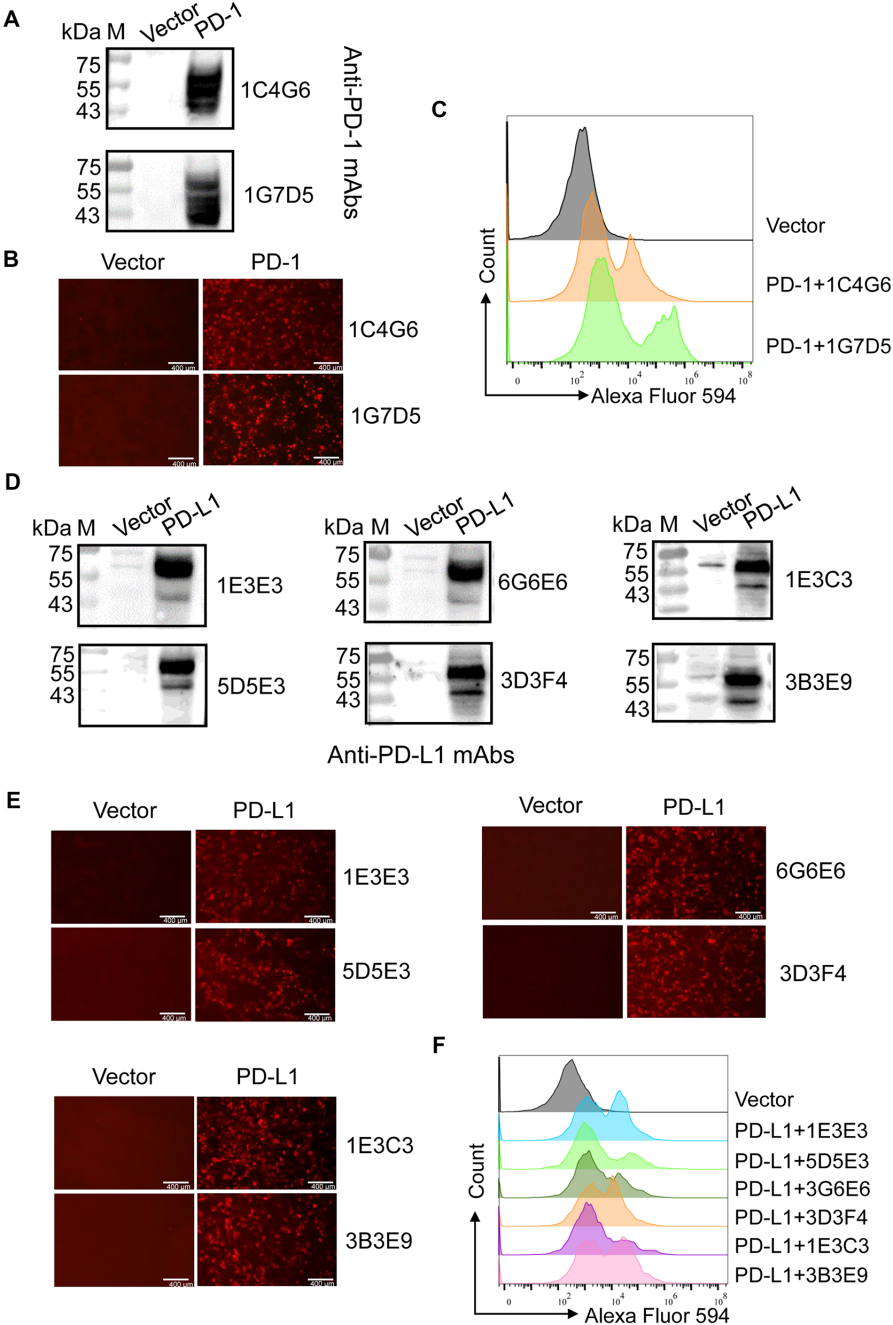
**Specificity identification of PD-1 and PD-L1 mAbs**. The HEK293T cells were transfected with pcDNA3.1 vector, which was performed as a control, and pcDNA3.1-PD-1 or pcDNA3.1-PD-L1 plasmids for 24 h. Cells were collected for western blot by using two PD-1 (1G7D5 and 1C4G6) or six PD-L1 (1E3E3, 3G6E6, 3D3F4, 1E3C3, 5D5E3, and 3B3E9) mAbs as primary antibodies (**A**, **D**), or were incubated with the two PD-1 or six PD-L1 mAbs for IFA (**B**, **E**) and FCM (**C**, **F**) assay.

To detect the specificity of PD-1 and PD-L1 mAbs, HA-tagged PD-1 and PD-L1 recombinant plasmids were transfected into HEK293T cells, and protein expression was verified by western blot. Subsequent immunoblotting demonstrated that all PD-1 and PD-L1 mAbs exhibited exclusive reactivity with lysates from transfected cells (Figures 3A,D). Furthermore, IFA (Figures 3B,E) and FCM (Figures 3C,F) results revealed that these mAbs reacted with the PD-1 or PD-L1 protein presented on the surface of the cells.

### Epitope identification for mAbs

**Figure 4:**
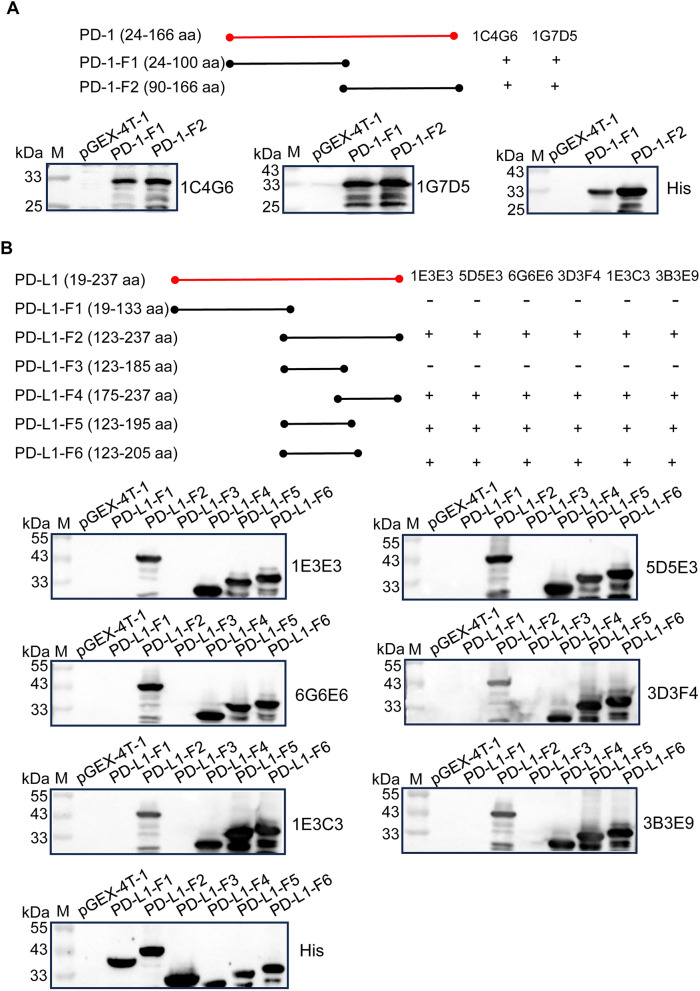
**Identification of B-cell epitopes recognized by the PD-1 or PD-L1 mAbs**. **A** Two overlapping truncated PD-1 fragments, PD-1-F1–PD-1-F2, were expressed in *E. coli* BL21 and subjected to western blot with 1C4G6,1G7D5 PD-L1 mAbs and His mAb; **B** Six overlapping truncated PD-L1 fragments, PD-L1-F1–PD-L1-F6, were expressed in *E. coli* BL21 and subjected to western blot with 1E3E3, 5D5E3, 6G6E6, 3D3F4, 1E3C3, 3B3E9 PD-L1 mAbs, and His mAb.

To characterize the epitopes recognized by mAbs, recombinant truncated fragments, two consecutive constructs share an overlap which consist of 10 amino acids, PD-1 and PD-L1 were expressed. According to the results in Figure 4A, PD-1 mAbs (1G7D5 and 1C4G6) bound to both truncated PD-1 proteins (PD-1-F1 and PD-1-F2). In Figure 4B, PD-L1 mAbs selectively bound to four of the six truncated PD-L1 proteins (PD-L1-F2, PD-L1-F4, PD-L1-F5, and PD-L1-F6), while showing no detectable reactivity with PD-L1-F1 and PD-L1-F3. All these results demonstrated that 1G7D5 and 1C4G6 mAbs recognized the epitope ^90^GRDPRFHVTPL^100^ of PD-1; 1E3E3, 3G6E6, 3D3F4, 1E3C3, 5D5E3 and 3B3E9 mAbs recognized the epitope ^185^REEKLFNVTST^195^ of PD-L1.

### Amino acid alignment and structural location of the epitopes

**Figure 5:**
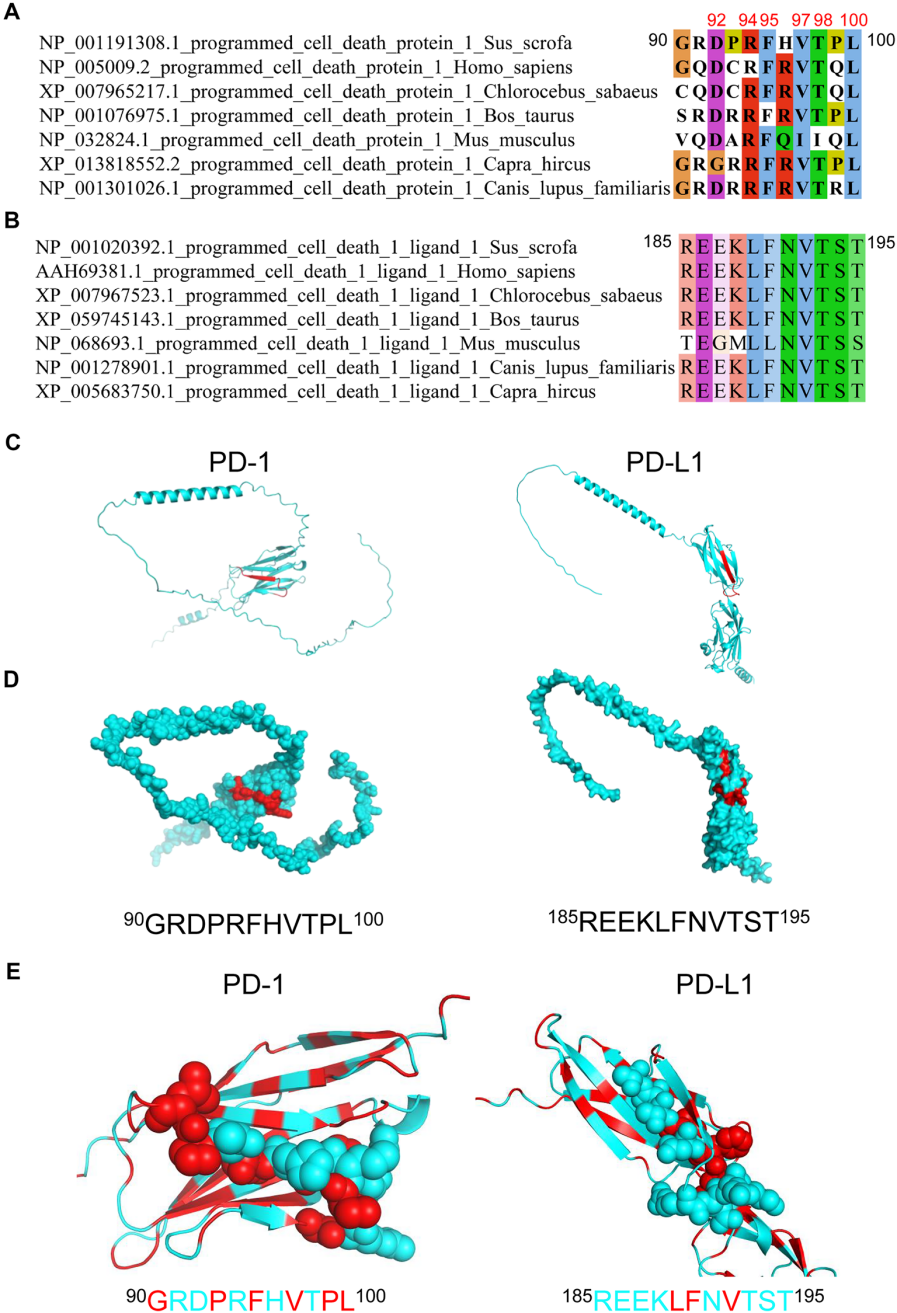
**Sequence alignment and spatial structure analysis of epitopes recognized by PD-1 and PD-L1 mAbs**. **A** Sequence alignment of ^90^GRDPRFHVTPL^100^ epitope in different species. The conservative amino acid residues of PD-1 cross species were labeled in red. **B** Sequence alignment of ^185^REEKLFNVTST^195^ epitope in different species. **C** and **D** In the predicted PD-1 and PD-L1 models, the B-cell epitopes were marked in red and shown as sphere (**C**) and cartoon (**D**). **E** Analysis of the hydrophilic and hydrophobic properties of epitope amino acids. Hydrophobic residues and hydrophilic residues of PD-1 and PD-L1 epitopes are, respectively, shown in red and cyan by spheres.

To characterize the recognition epitopes of PD-1 and PD-L1, we performed multiple sequence alignment across various species using Jalview software. Comparative sequence analysis demonstrated that the ^185^REEKLFNVTST^195^ epitope recognized by PD-L1 mAbs exhibited high evolutionary conservation, indicative of potential functional significance. In contrast, the ^90^GRDPRFHVTPL^100^ epitope of PD-1 merely displayed conservation at positions Asp-92, Arg-94, Phe-95, Val-97, Thr-98, and Leu-100 (Figures 5A, B).

To determine the structural localization of these epitopes, we predicted the tertiary structures of PD-1 and PD-L1 using AlphaFold3, and visualized them with Pymol 2.5.0 software. Structural analysis revealed that both epitopes reside in random coil regions, with ^90^GRDPRFHVTPL^100^ of PD-1 and ^185^REEKLFNVTST^195^ of PD-L1 forming part of a *β*-sheet and incorporating a short *β*-turn (Figure 5C). Furthermore, both epitopes were solvent-exposed, positioned on the protein surface, consistent with their role as potential B-cell recognition sites (Figure 5D). We further investigated the amino‑acid characteristics of the epitopes on PD‑1 and PD‑L1. The results showed that the epitopes recognized by the PD‑1 mAbs contain hydrophilic and hydrophobic residues that are scattered throughout the epitopes, whereas the epitopes recognized by the PD‑L1 antibody consist of consecutive hydrophilic residues (Figure 5E). Taken together, the PD-L1 epitope resides on the surface of the native PD-L1 protein, and exhibits almost 100% interspecies conservation, indicating that the identified epitope of PD-L1 mAbs is likely to be a valuable B-cell epitope.

### Assessment of mAbs blocking effect in vitro

**Figure 6:**
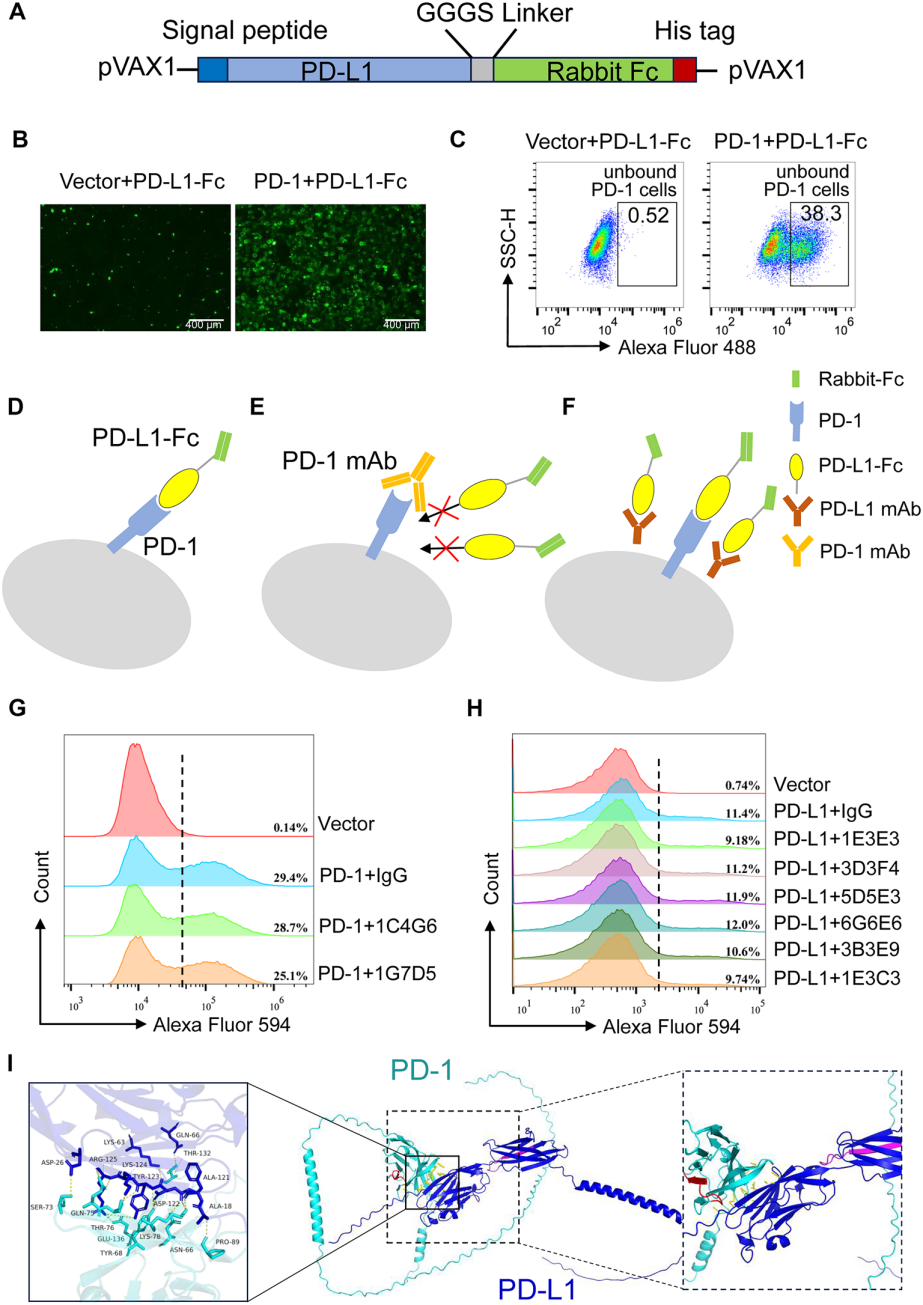
**Identification of blocking effect of PD-1 and PD-L1 mAbs. A** Schematic diagram of PD-L1-Fc recombinant protein. Porcine PD-L1 and rabbit-derived Fc fragment were connected together through a GGGS linker. **B** and **C** The HEK293T cells were transfected with pcDNA3.1 (Vector) or pcDNA3.1-PD-1 plasmids for 24 h, and then PD-L1-Fc protein was added and incubated for 1 h. Fluorescence signal was detected by fluorescence microscope (**B**) and flow cytometer (**C**). **D.** Schematic diagram showing PD-L1-Fc protein binds to PD-1. **E** and **F** Working model to evaluate the blockade effects of PD-1 mAb (**E**) and PD-L1 mAb (**F**). **G** and **H** The detection of the blocking effect of two PD-1 (**G**) or six PD-L1 (**H**) mAbs on the binding efficiency between PD-1 and PD-L1-Fc. The proportion of PD‑1^+^ cells recognized by PD‑L1‑Fc protein is displayed in the figure, with the dashed line indicating the threshold for PD‑1-PD‑L1‑Fc positive cells. Two independent experiments with similar results were performed. **I** Docking model showing the interaction between porcine PD-1 and PD-L1 protein. In the global schematic diagram on the right, the epitope of PD‑1 mAbs ^90^GRDPRFHVTPL^100^ was highlighted in red, while the epitope of PD‑L1 mAbs ^185^REEKLFNVTST^195^ was highlighted in magenta. Moreover, the relative spatial arrangement of the epitopes and the PD‑1-PD‑L1 interaction region was zoomed-in and shown on the right.

To evaluate the blocking effects of the aforementioned mAbs on PD-1–PD-L1 interaction, a recombinant fusion protein comprising PD-L1 and rabbit Fc (PD-L1-Fc) was constructed, with the component rabbit Fc as the reporter of blocking efficiency of the mAbs (Figures 6A–D). To maximize the likelihood of preserving native protein conformation and functionality, the recombinant fusion protein was expressed in a suspension culture system using HEK293F cells. The PD-L1-Fc recombinant protein had a molecular weight of 57 kDa, and the supernatant was subjected to protein purification with Ni–NTA agarose resin.

The IFA and FCM results revealed that PD-L1-Fc recombinant protein could recognize the cell surface-expressing PD-1 protein with a reaction rate around 38.3%, while with no specific reaction to cells transfecting empty vectors (Figures 6B, C and Additional file 1). To detect whether the binding between PD-1 and PD-L1 could be blocked by PD-1 or PD-L1 mAbs (Figures 6D–F), the cells or PD-L1-Fc protein were pretreated, respectively. As shown in Figure 6G, compared with the control, PD-1 mAb 1G7D5 treatment was associated with a mild 4% reduction in the interaction of PD-1 and PD-L1-Fc, while another PD-1 mAb had no effect. After pretreating PD-L1-Fc protein with PD-L1 mAbs 1E3E3 and 1E3C3 to mask its potential interacting sites with PD-1, a 2% reduction in the binding rate of PD-1 to PD-L1-Fc was observed in comparison with IgG control (Figure 6H). These data demonstrated that the PD-L1-Fc fusion protein provides a reliable tool for evaluating PD-1–PD-L1 blockade and facilitated the identification of three mAbs with partial blockade activity.

To further elucidate the structural basis of porcine PD-1–PD-L1 interaction and determine whether the identified epitopes overlap with functional interaction sites, we performed molecular docking simulations using AlphaFold3. Our data revealed that PD-L1 binds to PD-1 by forming hydrogen bonds with the ASN-66, TYR-68, SER-73, GLN-75, THR-76, LYS-78, PRO-89, and GLU-136 residues of PD-1 (Figure 6I). We marked the positions of epitopes in the interaction map of PD-1-PD-L1. The results revealed that the antigenic epitopes of PD-1 and PD-L1 are not located at the binding domain of the PD-1-PD-L1 interaction. These further confirmed that the forementioned PD-1 and PD-L1 mAbs cannot block the interaction of PD-1 and PD-L1 through competitive binding.

## Discussion

PD-1–PD-L1, as crucial immune checkpoint molecules, are key molecular targets for the immune evasion of pathogens. The activation of the PD-1–PD-L1 pathway can prevent the escalation of inflammation and reduce tissue damage. But at the same time, it also inhibits the activation of CD4^+^ and CD8^+^ T cells, causing them to undergo apoptosis, suppress the host’s immune response, and lead to immune evasion by tumors or pathogenic microorganisms. A list of animal diseases, such as PRRS, mycoplasma hyopneumoniae, Glässer’s disease, and echinococcosis, are associated with host immune suppression [18]. Bovine leukemia virus (BLV) is known to induce immunosuppression and B cell lymphoma in cattle, and lead to enormous damages to livestock industries around the world [19]. Studies have confirmed that PD-1 or PD-L1 blockade by anti-PD-1 mAb could upregulate the immune reaction during chronic infection, and downregulate the BLV provirus load in bovine PBMCs [20, 21]. Goto et al*.* reversed T-cell exhaustion induced by the bovine mycoplasma via PD-1–PD-L1 blockade [22]. After treatment with a blocking anti‑PD‑L1 antibody, PBMCs from infected pigs were able to restore the interleukin (IL)‑2 production [4]. Therefore, PD-1–PD-L1 blockade by mAbs could provide a new therapy to control pathogen infection via activating immune response.

To date, no studies have reported the application of PD-1–PD-L1 blockade in swine disease models, nor have a working platform to identify blocking mAbs or epitopes of PD-1 or PD-L1 for animal host. To address these issues, recent studies have aimed to obtain and screen excellent antibodies based on the immune checkpoint PD-1 and PD-L1 protein to develop new diagnostic tools. The technology of producing recombinant proteins using prokaryotic and eukaryotic expression (*E. coli*, yeast, insect baculoviruses, transgenic plants, and mammalian cells) host systems has paved the way for the development of vaccines and antibodies [23]. In the present study, pET32a-PD-1 and pET32a-PD-L1 recombinant protein were induced via *E. coli* prokaryotic expression system because of its high expression output, easy procedure, and low cost. PD-L1-Fc recombinant protein was obtained with excellent purity by mammalian cells owing to the high requirement for the native confirmation of the protein. In view of the characteristics of the PD-1 and PD-L1 protein, after changing the temperature of induction, concentration of IPTG and vector, we finally purified PD-1 and PD-L1 protein with high purity from the precipitate of bacterial lysate after induction. The mice produced high antibody titers against the PD-1 or PD-L1 protein after being immunized with recombinant proteins three times, which lay a foundation for subsequent mAbs preparation.

Since the inception of hybridoma technology, monoclonal antibody technology has rapidly evolved, providing novel approaches for the diagnosis, prevention, and treatment of clinical diseases. The primary techniques for obtaining monoclonal antibodies currently include hybridoma technology, phage display antibody library technology, and single B-cell antibody technology, among others. In this study, hybridoma technology, which is based on cell fusion techniques to combine sensitized B cells with the ability to secrete specific antibodies with SP2/0 cells that possess the capacity for unlimited proliferation, was utilized for the preparation of mAbs. This fusion results in the formation of hybridoma cells that can both secrete antibodies and be passaged indefinitely. Finally, several homogeneous hybridoma cell lines capable of stable antibody secretion were obtained after three rounds of subcloning, including two mAbs against porcine PD-1 and six against porcine PD-L1 proteins. The stability and high titer of hybridoma cells and mAbs have been confirmed by ELISA, and the heavy and light chain types of all mAbs have been characterized. Further results revealed that all mAbs specifically recognize the natural PD-1 or PD-L1 proteins by western blot, IFA, and FCM, suggesting that the generated PD-1 or PD-L1 mAbs serve as reliable research tools for exploring the detection of immune status and facilitating the development of clinical application techniques.

The rapid progress in proteomics and bioinformatics has led to the development of diverse methodologies for antigenic epitope identification, ranging from conventional peptide synthesis [24] and peptides scanning [25, 26] to advanced techniques such as mass spectrometry [27] and bioinformatics prediction. In this study, the antigenic epitopes of PD-1 and PD-L1 mAbs were recognized using truncated protein expression method. The ^90^GRDPRFHVTPL^100^ of PD-1 and ^185^REEKLFNVTST^195^ of PD-L1 were identified by truncating PD-1 and PD-L1 protein. Amino acid sequence alignment showed that antigenic epitopes of PD-1 had conserved sites at positions Asp-92, Arg-94, Phe-95, Val-97, Thr-98 and Leu-100, and that of PD-L1 was completely conserved among different species, which suggested that our PD-L1 mAbs may have good broad-spectrum recognition ability. Further, spatial location of the epitopes analysis revealed that the two identified epitopes were fully exposed on the surface of PD-1 or PD-L1 proteins, maximizing the possibilities for antigen–antibody binding and likely representing valuable B-cell epitopes.

Fc-fusion proteins are composed of Fc region of IgG antibody (Hinge-CH2-CH3) and a desired linked protein. Fc region of Fc-fusion proteins can bind to neonatal Fc receptor (FcRn) thereby rescuing it from degradation [28]. At present, Fc fusion proteins have been widely applied in the fields of detection, therapy, and vaccine development [29–31]. In this study, we utilized HEK293F cells to express PD-L1-rabbit Fc fusion protein in vitro, successfully binding to and visualizing PD-1 molecules on the cell surface. After treatment with the PD-1 mAbs 1G7D5, the binding efficiency of the PD-L1-Fc protein to PD-1 was reduced by 4%, and PD-L1 mAbs 1E3E3 and 1E3C3 exhibited only a 2% blocking effect in comparison with IgG control. The interaction of ligand–receptor can be directly interrupted when the conformational epitope of an antibody overlaps the receptor-binding pocket of protein. A respiratory syncytial virus (RSV) F protein antibody D25, which targets the ^Ø^ epitope of prefusion and occludes the receptor site of F protein, exhibits a blocking potency around 50-fold greater than that of the clinically approved palivizumab antibody [32]. Based on the results of molecular docking, we found that the binding sites of the mAbs obtained in this study are not located in the interaction sites between porcine PD-1 and PD-L1 protein. This may explain the poor blocking ability of the mAbs. The hydrophobicity or hydrophilicity of an antibody epitope profoundly shapes affinity and blocking potency. Hydrophobic patches confer high affinity, whereas they often trigger conformational “collapse” that reduces blocking efficiency. In contrast, the binding affinity of the hydrophilic peptide segment is moderate, yet its blocking efficacy is superior. Zhong et al. identified an excellent PD‑L1 competitive‑binding peptide L7, which could bind directly to the PD-1–PD-L1 IgV domains. Hydrophobic amino acids constitute a relatively large proportion in L7 (REEKLFNVTSTLRINT) and are distributed continuously. By substituting the nonconserved hydrophobic residues (Phe^6^ and Thr^11^) with hydrophilic residues, they increased the peptide’s aqueous solubility while preserving its functional activity [33]. In our study, the epitope recognized by PD‑1 mAbs comprises an equal proportion of hydrophilic and hydrophobic amino acids, which are interspersed. This heterogeneous distribution may affect the binding affinity between the antibody and the PD-1 protein, thereby influencing the blocking efficacy. In contrast, the epitope recognized by PD‑L1 is dominated by contiguous hydrophilic residues, indicating that the PD‑L1 antibody exhibits a stronger affinity for the PD-L1 protein. We propose that the lack of any overlap between the peptides and the PD‑1–PD‑L1 binding interface is a key factor underlying the low blocking efficiency. Although the PD‑1–PD‑L1 mAbs obtained in this study lack strong blocking activity, their excellent reactivity and sensitivity make them highly valuable for clinical applications. The epitopes identified here might be used to screen swine populations or other host species for individuals with high PD-1 or PD‑L1 expression, thereby guiding the therapeutic strategies; moreover, they enable effective monitoring of PD‑1–PD‑L1 dynamics during treatment, facilitating assessment of therapeutic efficacy and immune responses. On the other hand, for the development of blocking antibodies in future, the interaction region of PD-1 and PD-L1 protein can be targeted for antigen design, which may contribute to improving the blocking efficacy.

In summary, we prepared eight monoclonal antibodies against porcine PD-1 and PD-L1 proteins, and identified two unreported linear B-cell epitopes on PD-1 and PD-L1 mAbs. More importantly, based on the porcine PD-L1-rabbit Fc fusion protein, we herein established a powerful tool for the screening of PD-1–PD-L1 blocking antibodies, providing meaningful tools for diagnostic and therapeutic purposes.

## Supplementary Information

**Additional file 1 Gating strategy for detecting free PD‑1 protein on the cell surface. **Based on the FSC‑SSC plot, the total target cell population was gated. Single cells were then selected using FSC‑A and FSC‑H parameters for downstream analysis. By employing Alexa Fluor 488‑conjugated PD‑L1‑Fc, which binds to surface PD‑1, the proportion of cells displaying surface PD‑1 could be quantified under the Alexa Fluor 488 positive gate.

## Data Availability

The data used and/or analyzed during the current study are available from the corresponding author upon reasonable request.
