# Supplementary figures and images for: Identification of PD-1–PD-L1 blockade epitopes in vitro utilizing porcine immunoglobulin and heterologous Fc-fused protein
Source: Vet Res. 2025 Nov 27;56:236. doi: 10.1186/s13567-025-01674-x (PMC12752125; doi:10.1186/s13567-025-01674-x)

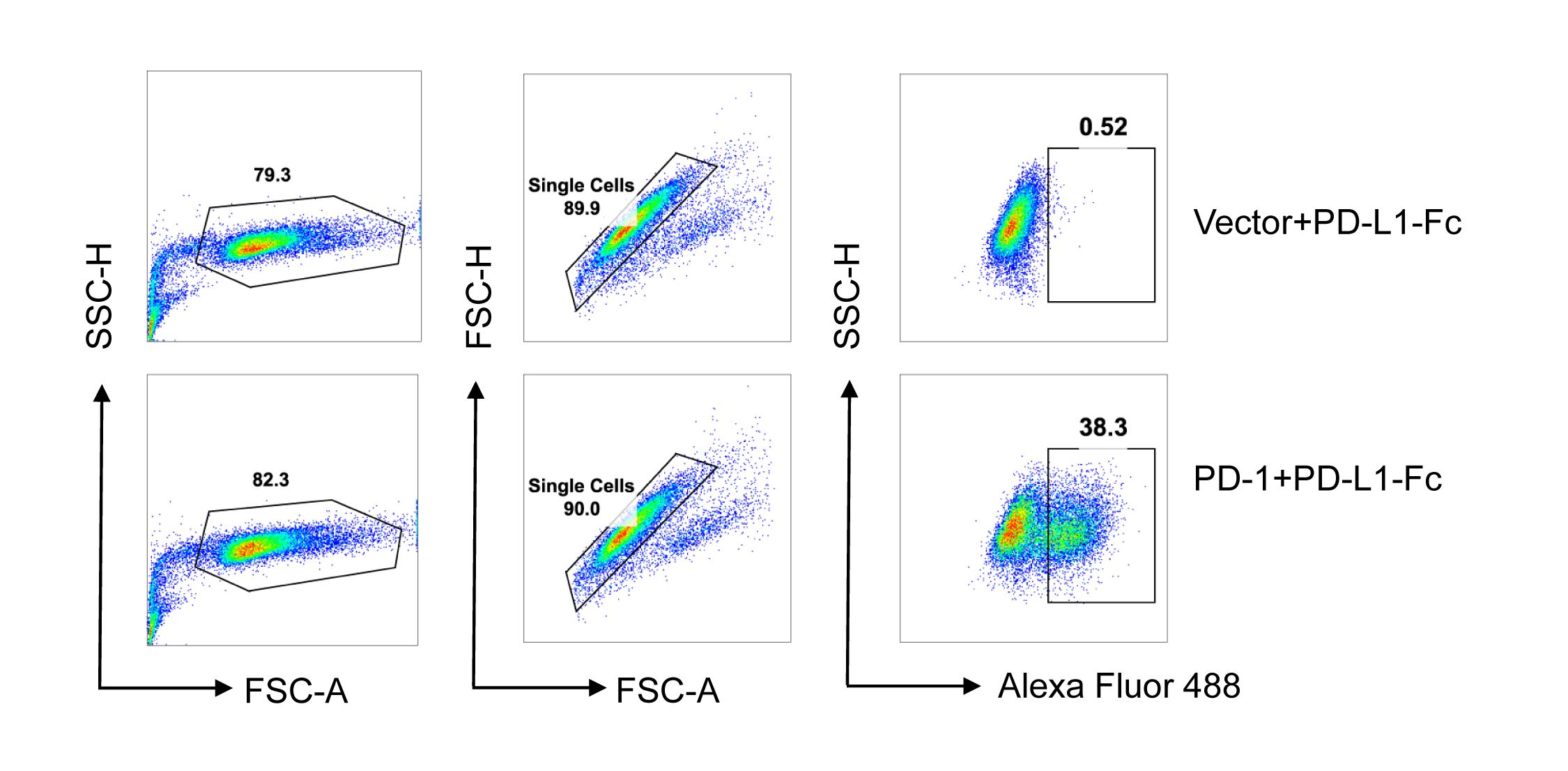

Supplement: Supplementary file 1 — Additional file 1 Gating strategy for detecting free PD‑1 protein on the cell surface. Based on the FSC‑SSC plot, the total target cell population was gated. Single cells were then selected using FSC‑A and FSC‑H parameters for downstream analysis. By employing Alexa Fluor 488‑conjugated PD‑L1‑Fc, which binds to surface PD‑1, the proportion of cells displaying surface PD‑1 could be quantified under the Alexa Fluor 488 positive gate. [file 13567_2025_1674_MOESM1_ESM.tif]
